# Differential producibility analysis reveals drug-associated carbon and nitrogen metabolite expressions in *Mycobacterium tuberculosis*

**DOI:** 10.1016/j.jbc.2025.108288

**Published:** 2025-02-08

**Authors:** Ye Xu, Ruma Banerjee, Sunitha Kasibhatla, Johnjoe McFadden, Rajendra Joshi, Khushboo Borah Slater

**Affiliations:** 1 Department of Clinical Laboratory, Shanghai Ninth People’s Hospital, Shanghai Jiao Tong University School of Medicine, Shanghai, China; 2 School of Biosciences, University of Surrey, Guildford, United Kingdom; 3 High Performance Computing: Medical & Bioinformatics Applications Group, Centre for Development of Advanced Computing (C-DAC), Pune, India; 4 Faculty of Health and Life Sciences, Department of Biosciences, University of Exeter, Exeter, United Kingdom

**Keywords:** antibiotics, bacterial metabolism, *Mycobacterium tuberculosis*, tuberculosis

## Abstract

*Mycobacterium tuberculosis* (Mtb) is one of the world’s successful pathogens that flexibly adapts its metabolic nature during infection of the host, and in response to drugs. Here we used genome scale metabolic modelling coupled with differential producibility analysis (DPA) to translate RNA-seq datasets into metabolite signals and identified drug-associated metabolic response profiles. We tested four tuberculosis (TB) drugs bedaquiline (BDQ), isoniazid (INH), rifampicin (RIF), and clarithromycin (CLA); conducted RNA-seq experiments of Mtb exposed to the individual drugs at subinhibitory concentrations, followed by DPA of gene expression data to map up and downregulated metabolites. Here we highlight those metabolic pathways that were flexibly used by Mtb to tolerate stress generated upon drug exposure. BDQ and INH upregulated maximum number of central carbon metabolites in glycolysis, pentose phosphate pathway and tri-carboxylic acid cycle with concomitant downregulation of lipid and amino acid metabolite classes. Oxaloacetate was significantly upregulated in all four drug-treated Mtb cells highlighting it as an important metabolite in Mtb’s metabolism. Amino acid metabolism was selectively induced by different drugs. We have enhanced our knowledge on Mtb’s carbon and nitrogen metabolic adaptations in the presence of drugs and identify metabolic nodes for therapeutic development against TB. Our work also provides DPA omics platform to interrogate RNA-seq datasets of any organism that can be reconstructed as a genome scale metabolic network.

Tuberculosis (TB) continues to pose a significant global health challenge, retaining its status as one of the leading causes of mortality from a single infectious agent *Mycobacterium tuberculosis* (Mtb). Mtb infects over a quarter of the global population, resulting in approximately 10.8 million cases and 1.25 million deaths (1). The burden of TB is exacerbated by other health concerns, such as HIV, smoking, poverty, diabetes, and malnutrition (1). Moreover, TB imposes a considerable economic and financial burden, costing over 13 billion dollars annually (1, 2). Despite advancements in TB treatment that reduced the mortality rate from over 50% to less than 15%, the emergence of drug-resistant (DR) TB has made treatment increasingly challenging (1). The current standard regimen for drug-sensitive TB involves a 6-month treatment with rifampicin (RIF), isoniazid (INH), pyrazinamide, and moxifloxacin (1). However, DR-TB requires the use of second-line drugs, including fluoroquinolone, bedaquiline (BDQ) or linezolid for up to 36 months (1, 2). The prolonged application of second-line drugs is associated with substantial expenses and side effects, potentially exacerbating the emergence of multidrug-resistant TB (MDR-TB) (3). Unfortunately, resistance to BDQ, pretomanid, or delamanid has been documented in clinical settings (4, 5, 6), which further limits the treatment options. The prolonged duration, toxicity, and costs of the drugs significantly compromise patients' quality of life, amplifying non-compliance (7, 8, 9). Besides drug resistance, drug tolerance or persistence also contributes to treatment failure, further limiting the efficacies of current treatments (3, 10, 11, 12).

Therefore, to avoid unfavorable outcomes in TB, it is crucial to thoroughly understand the bactericidal processes of drugs. The bactericidal activities of drugs is not only mediated by direct target inhibition but also through the downstream biological processes that amplify the effects, ultimately leading to the death of Mtb. For instance, the bactericidal effect of INH is not only mediated by inhibition of mycolic acid biosynthesis (10) but also mediated by hindering overall fatty acid biosynthesis and induction of reactive oxygen species (13, 14). Similarly, for BDQ, inactivation of the F_0_F_1_ ATP synthase not only prevents energy production but also inhibits glycogenolysis and shifts the tricarboxylic acid (TCA) cycle to the methyl citrate cycle to produce toxic intermediates, which ultimately leads to cell death (15).

Mtb has developed metabolic flexibility to withstand biological perturbation induced by environmental stresses, like drugs. Mtb has a fully functional TCA cycle, glycolysis, gluconeogenesis, pentose phosphate pathway (PPP) and the methyl citrate cycle, which enable the refueling of metabolic intermediates and reprogramming of fluxes to sustain stress and nutrient limiting conditions such as in the host environment (16). In response to INH induced-reactive oxygen species, Mtb can downregulate NADH dehydrogenase by switching from the use of the TCA cycle to glyoxylate shunt (17). Similarly, in the presence of BDQ, Mtb shifts from glycolysis to the PPP in order to reduce the ATP consumption at the phosphofructokinase step and generates substrate-level ATP *via* pyruvate kinase, resulting in delayed bactericidal effects of BDQ (15, 18).

Despite Mtb’s flexibility to withstand drug-induced changes, the compensatory pathways or metabolic modulations that allow Mtb to tolerate and survive in the presence of drugs are vulnerable targets for therapeutic development. For instance, the dependence on glycolysis-generated ATP in BDQ-treated Mtb makes it susceptible to the disruption of glycolysis or gluconeogenesis, indicating potential synergistic treatment by combining BDQ with inhibitors on the glycolysis pathway to rapidly sterilize Mtb (15). Likewise, INH-treated Mtb inactivates *katG* to survive treatment, paradoxically rendering Mtb susceptible to oxidative stress (19). Thus, the vulnerabilities present an opportunity for devising combination treatments.

The crucial role of metabolism in Mtb’s physiology drives the necessity of systemically evaluating metabolic regulations. Genome-scale metabolic modeling offers a cost-effective and efficient way to interpret metabolic status systematically without requiring sophisticated laboratory equipment, such as mass spectrometers. Especially, by integrating large datasets, like ‘omics’ data, into these models, the metabolic state of Mtb across various experimental conditions can be accurately predicted (20). Differential producibility analysis (DPA) is one of the computational platforms combining genome-scale modeling and flux balance analysis (FBA) to interrogate transcriptomics data for quantitative assessment of changes in metabolite production rates across different physiological conditions (21). DPA utilizes the systems-wide knowledge of enzyme-encoding genes to build the gene-metabolic reactions network and determines gene essentiality in metabolites’ production based on FBA (21). The later incorporation of transcriptome (RNA-seq) data under various conditions can accurately present metabolic shifts in the organism of interest. In Mtb, the introduction of the first genome-scale metabolic models GSMN-TB or iNJ661 laid the foundation for predicting phenotype and gene essentiality during *in vitro* growth (22, 23). Over time, these models were refined with further annotation of the metabolic genes, broadening their prediction capability to encompass physiological phenotype during *in vivo* growth or host infection (24, 25, 26, 27, 28, 29).

In this study, we employed DPA to interrogate RNA-seq datasets to understand the metabolic adaptations of Mtb required to survive in the presence of various drugs: INH, RIF, BDQ, and clarithromycin (CLA). The knowledge of Mtb’s metabolic adaptation to drugs could help us understand the key metabolites and pathways that are crucial for Mtb to tolerate drug-induced stress and may provide new targets for combinatorial anti-TB chemotherapies.

## Results

### Genome scale modeling to assess growth of Mtb on various drugs

**Figure 1:**
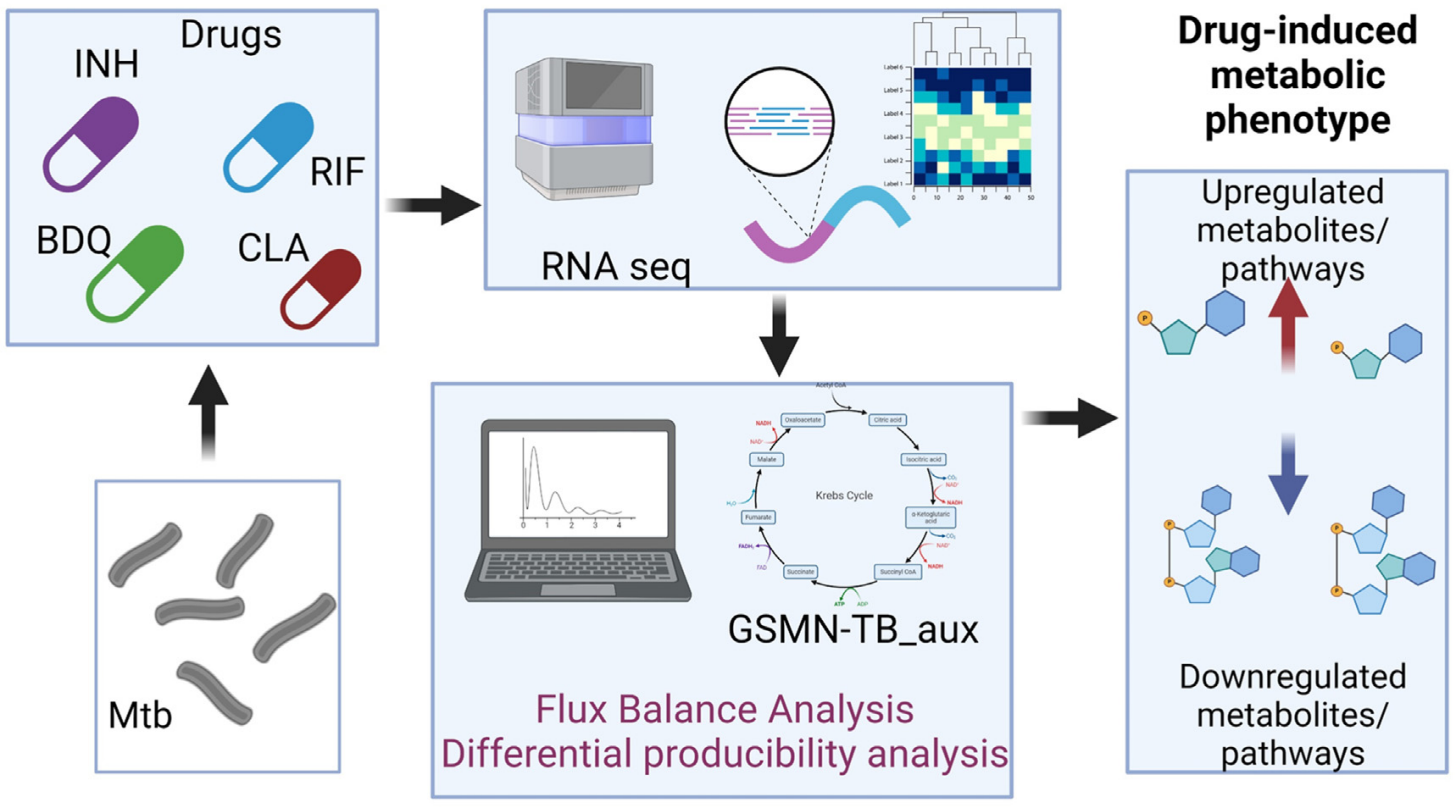
**Schematic of experimental and computational workflow for differential producibility analysis (DPA).** INH (isoniazid), RIF (rifampicin), BDQ (bedaquiline), and CLA (clarithromycin) were used at one-fourth MIC to treat Mtb H37Rv mc^2^6260 for 24 h. RNA-seq was performed after antibiotic treatment (31). Producibility plots are calculated using FBA and knockout analysis using GSMN-TB_aux which were mapped to the RNA-seq data for drawing up a list of up and down-regulated metabolites.

We used our genome scale metabolic model: GSMN-TB_2 as a reference to construct GSMN-TB_aux representing the Mtb strain mc^2^6260 which is auxotrophic for leucine and pantothenate (supplementary file 1). Reactions: R176, R392, and R221 corresponding to the genes *Rv3601c* (*panD*), *Rv3602c* (*panC*), *Rv2987c* (*leuD*), and *Rv2988c* (*leuC*) for pantothenate and leucine biosynthesis were removed from the GSMN-TB_2 model and uptake reactions for pantothenate and 3-carboxy-2-hydroxy-4-methylpentanoate (CBHCAP) were introduced into the model for uptake of leucine and pantothenate. The uptake reactions were opened in the media file. Glycerol was the main carbon source and additional components such as tween-80, citrate, phosphate, glutamate, biotin, cobalt, iron, sulfate was added to replicate the 7H9 media as used in the experimental conditions. FBA was performed using the ‘sfba-glpk’ solver available in SurreyFBA2.3 package (30). DPA was performed to convert RNA-seq data into metabolite expression profiles for identification of significantly up or downregulated metabolites in Mtb exposed to the four antibiotics (Fig. 1).

To identify metabolic changes associated with the drug treatment, Mtb cells were grown in the presence of drugs: INH, CLA, RIF and BDQ as independent experiments using one-fourth MIC of the drugs (see methods for details). A subinhibitory concentration of one-fourth MIC was selected based on previous findings (31), which demonstrated that one-fourth MIC was the highest concentration with minimal effects on bacterial growth over a period of 2 days (Fig. S1). However, it was sufficient to effectively kill Mtb in combination treatments exhibiting antibacterial effects as shown in the time-kill assays (Fig. S2 (31)). Therefore, subinhibitory concentrations were used to measure the active metabolic responses of Mtb to drug treatment without inducing extensive stress responses. For Fig. S1, the ratio is derived by comparing the CFUs of Mtb under indicated drugs at sub-inhibitory concentration to the CFUs of starter inoculums of 10^7^ CFU/ml. The ratio was >1 for drug treatment at the sub-inhibitory concentration, as it would not inhibit bacterial growth fully, and therefore, was chosen as the drug concentration for subsequent RNA-seq experiments.

**Figure 2:**
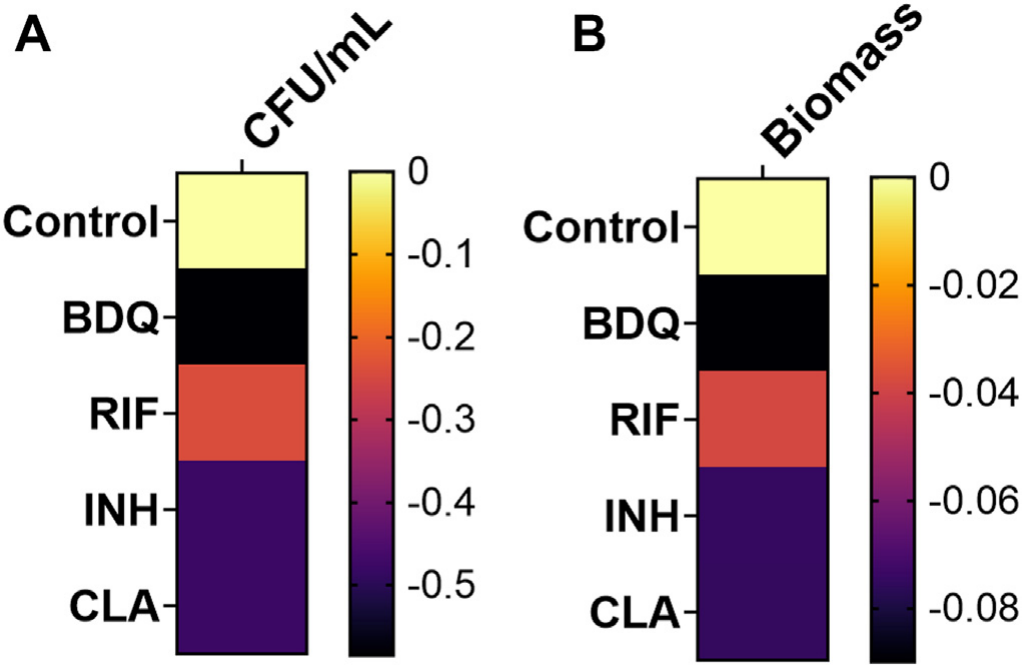
**Biomass production by GSMN-TB_aux.***A*, Heat map showing the log 2-fold change of CFUs of Mtb exposed to drugs vs. the control (untreated/no drug) condition. CFUs were calculated from the average of three biological replicates. *B*, Heat map showing the log 2-fold change of biomass production calculated using FBA of GSMN-TB_aux in growth media with various drugs. Data are shown for the four drugs compared to the control (untreated/no drug) condition. The threshold for log 2-fold change was set to ≤0.1 and ≤ 0.01 for the CFU and biomass respectively to capture any differences between the control and treated cells. The log 2-fold changes for CFUs and biomass were reduced in the presence of drugs with BDQ exhibiting the maximum reduction. CFUs and biomass are shown for Mtb exposed to one-fourth MICs of BDQ, RIF, INH, and CLA using 7H9 as the base media (see methods for details for *in silico* media formulation).

We validated the *in silico* biomass production by GSMN-TB_aux on each of the four drugs by comparing it to the CFU of Mtb treated with the same drugs for 24 h (Fig. 2). The heat maps in Figure 2 shows the log 2-fold change of biomass and CFUs compared to the control (untreated). The biomass production patterns were identical to that of the CFU counts of Mtb when treated with each of the drugs, *i.e.*, BDQ > CLA > INH > RIF with BDQ treatment resulting in the maximum reduction of CFU and biomass production (highest negative log fold-change). We also simulated the biomass production with GSMN-TB_2 original Mtb model in the presence of the four drugs to crosscheck the robustness of GSMN-TB_aux predictions. The predictions for drug associated biomass productions by GSMN-TB_2 followed the same pattern as the CFUs measured, and the biomass production by GSMN-TB_aux (reduction in CFU and biomass: BDQ > CLA > INH > RIF) (Fig. S3) validating the use of the auxotrophic strain as a model for Mtb wild-type strain, and the model GSMN-TB_aux to accurately predict experimentally relevant drug-phenotype. These tests were important to check the reliability of the model for subsequent DPA that used computational modelling to map the RNA-seq datasets into metabolite production profiles. DPA generated metabolite expression profiles identified the adaptations that Mtb employs to survive in presence of the subinhibitory concentration of the drugs.

### DPA analysis of Mtb under various drugs

**Figure 3:**
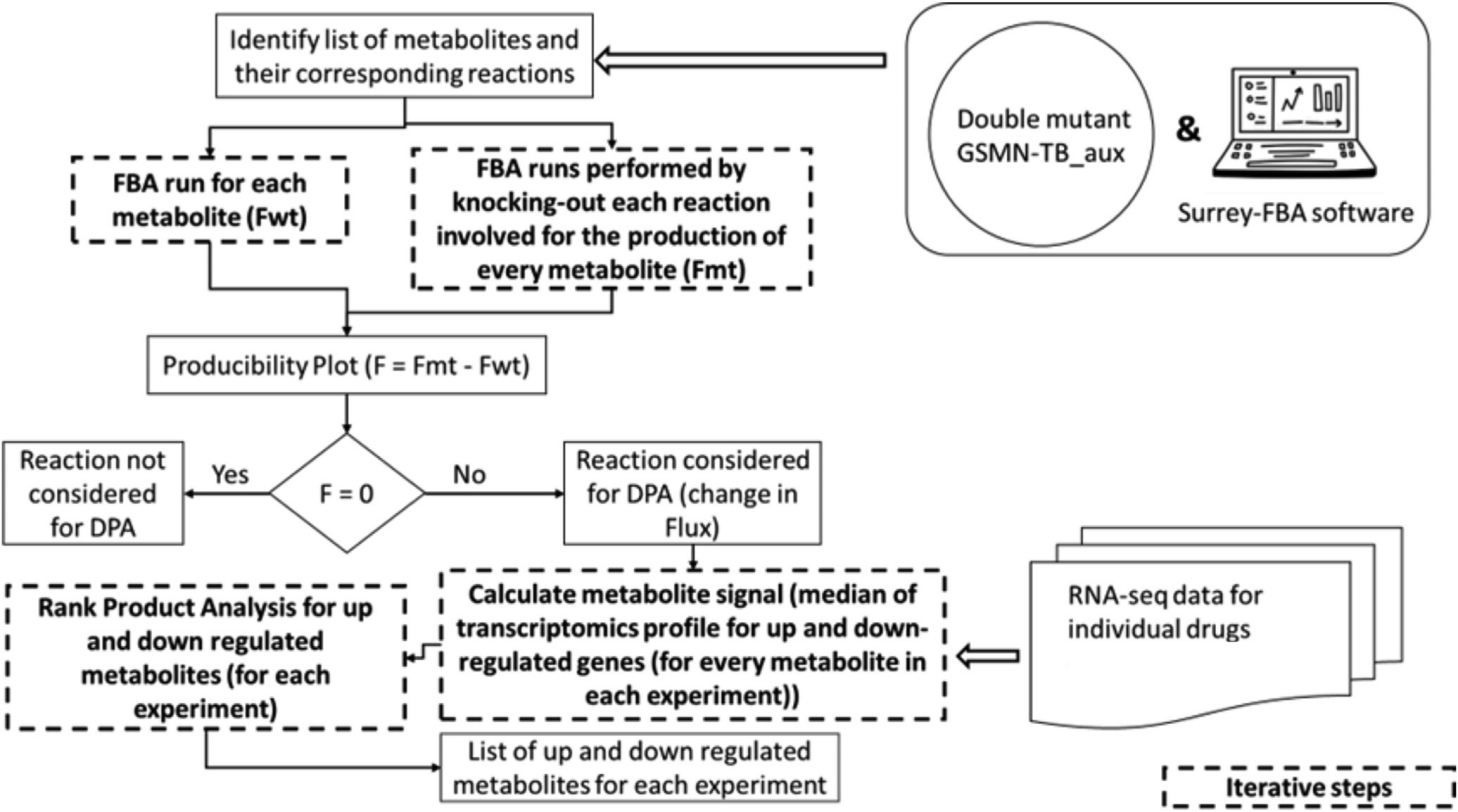
**Schematic representation of the steps performed in Differential Producibility analysis (DPA).** The workflow shows step-by-step analysis starting with flux balance analysis (FBA) to generate a producibility plot for metabolites that are up and downregulated under various conditions. RNA-seq gene expression profiles were mapped to the reactions producing the corresponding metabolites. Rank product analysis was used to draw up the final list of up- and downregulated metabolites in Mtb exposed to various drugs.

DPA allowed us to calculate metabolite abundance values from their corresponding differentially expressed genes using the GSMN-TB_aux genome scale metabolic network. Genes participating in the production of every metabolite were identified using FBA where the objective function was set to maximize the metabolite production in the wild type (Fwt). The process is iterated over all metabolites in the network (Fig. 3). Subsequently, knock-out analysis for each metabolite was performed, in which, systematic deletion of one reaction at a time (involved in the metabolite production) is done followed by calculation of rate of change in flux using FBA in the mutant (Fmt). This step is also iterated over all metabolites in the network. The difference in flux (ΔF) for each reaction in a metabolite was calculated using the formula:

ΔF = Fmt – Fwt;

The difference in fluxes for a reaction between the mutant and the wild type is a matrix; where the rows are metabolites, and the column are genes. The non-zero ΔF values are the reactions which are essential to produce a given metabolite and a value of “1” is assigned in the matrix. The binary form of the matrix is visualized as a dot-plot and is referred to as the “Producibility Plot”. The metabolite signals were calculated as the median of the gene(s) expression values (involved in the production of a given metabolite) obtained from RNA-seq experiments performed to study the effect of drugs on Mtb. This analysis was conducted separately for up- and down-regulated genes. Each metabolite was then ranked based on the average intensity of the transcriptome profile associated with the genes influencing its production, using non-parametric two class rank analysis. The up and down-regulated metabolites for each experiment were further analyzed to understand the effect of drugs along with substrate utilization and its mechanism towards persistence in Mtb.

### BDQ and INH modulated maximum number of metabolites

**Figure 4:**
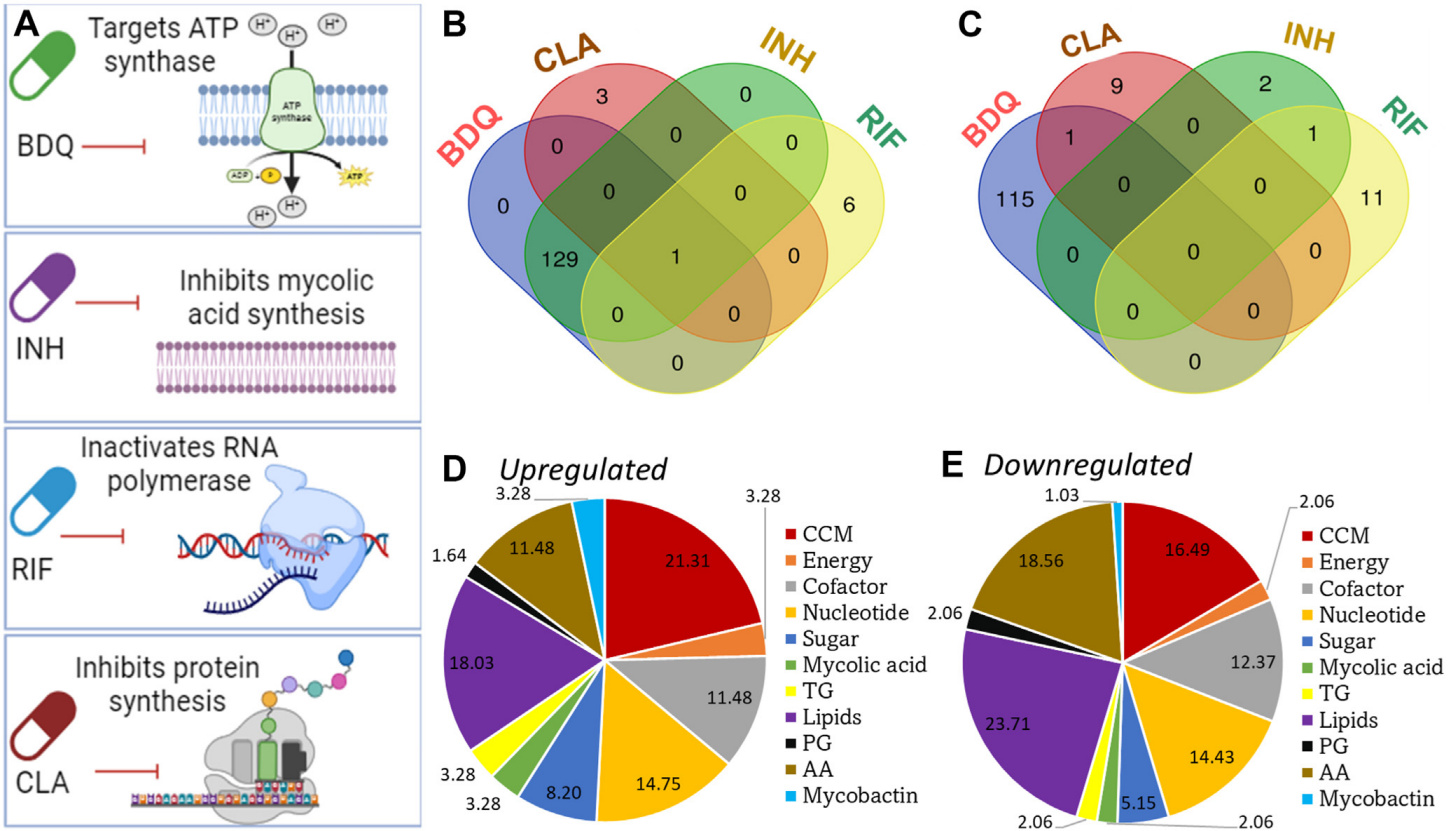
**DPA profiles for Mtb exposed to various drugs.***A*, molecular and cellular targets for bedaquiline (BDQ), isoniazid (INH), rifampicin (RIF) and clarithromycin (CLA). *B*, Venn diagram representation of metabolite classes upregulated in Mtb in the presence of one-fourth MICs of BDQ, INH, RIF and CLA. *C*, Venn diagram representation of metabolite classes downregulated in Mtb in the presence of one-fourth MICs of BDQ, INH, RIF and CLA. *D*, proportional distribution (%) of upregulated metabolites grouped in BDQ and INH. These metabolites are commonly upregulated in BDQ and INH. *E*, proportional distribution (%) of downregulated metabolites in BDQ. Metabolites were grouped into central carbon metabolism (CCM), energy, cofactor, nucleotide, sugar, mycolic acids, triglycerides (TG), lipids, peptidoglycan (PG), amino acids (AA) and mycobactin pathways to simplify presentation. DPA datasets were generated using experimental RNA-seq data from three biological replicates of Mtb cultures grown in the presence of drugs for 24 h.

BDQ, INH, RIF and CLA have established mechanism of actions for killing Mtb. BDQ targets ATP synthase; INH inhibits mycolic acid synthesis; RIF targets RNA polymerase and CLA inhibits protein synthesis (Fig. 4*A*). However, the effects of these drugs on the central metabolism of Mtb have not been fully understood. This knowledge will help us identify how Mtb adapts its metabolism in response to drugs for its survival and highlight any additional target(s) for these drugs that could help us design new synergistic drug combinations for effective and rapid killing of Mtb. Here we used DPA analysis to identify and rank up and downregulated metabolites in Mtb when exposed to the subinhibitory concentrations of these drugs (Fig. 4, *B* and *C*). We focused on the central carbon and nitrogen metabolites to identify if there are any metabolites in these central pathways that are specifically targeted by various drugs (described in the next section). The elevated or depressed expression of these metabolites suggest their involvement in Mtb to withstand or survive in the presence of drugs. The ranking and cut off *p*-values for the up- and downregulated metabolite classes are included in supplementary file 2. BDQ and INH upregulated the maximum number of metabolites (129) that are common in both drugs (Fig. 4*B*). Only one metabolite: oxaloacetate (OAA) was commonly upregulated in all four antibiotics. OAA is an important metabolic intermediate involved in gluconeogenesis, anaplerosis and TCA cycle pathways which are functionally critical pathways for Mtb to maintain its virulence and survival. OAA has been demonstrated to be important for resistance and persister cell formation in *Escherichia coli*; in Mtb, OAA has been demonstrated to support growth in acidic pH and survival in macrophages (32, 33, 34). Hence, the upregulation of OAA reveals a cellular metabolic response that was common across all four drugs which had distinct mechanism of action(s).

Metabolites participating in central carbon metabolism (CCM) including glycolysis, TCA cycle and PPP were the most upregulated classes in BDQ and INH treated cells accounting for 21.3% (Fig. 4*D*). RIF and CLA had the least proportion of metabolites that were upregulated in Mtb in exposure to the sub-inhibitory concentration of the drugs; these classes primarily included metabolites involved in lipid and nucleotide biosynthesis and breakdown. Out of the four drugs tested, BDQ downregulated maximum proportion of metabolites (Fig. 4*C*). In contrast to the upregulated classes, BDQ and INH shared no common metabolite that was downregulated in both drugs. BDQ/CLA and INH/RIF had one metabolite downregulated in the presence of both drugs. Figure 4*E* shows the proportional distribution of metabolite classes that were downregulated in BDQ only. Lipids (including fatty acids and phosphatidylinositol mannoside (PIM)) and amino acid (AA) metabolite classes were the most downregulated classes, accounting for 23.7% and 18.5% respectively in BDQ-treated cells. Out of the four drugs tested, BDQ and INH were the two drugs which elicited maximum changes in metabolite expressions. RIF and CLA exhibited maximum inhibition of Mtb’s growth at one-fourth MIC compared to BDQ and INH. The survival ratios being lowest in RIF and CLA highlights lower biomass production which might have affected the number of molecular identities detected by RNA-seq and could provide a possible explanation for the fewer metabolites derived by DPA. RIF and CLA targeted RNA and protein synthesis that rapidly diminished cellular metabolic activity; hence the number of up and downregulated metabolites captured by DPA were limited compared to INH and BDQ. Mtb cells that survived on subinhibitory concentrations of BDQ and INH exhibited increased expression of CCM metabolites with concurrent downregulation of lipids and amino acids classes. Overall, we see drug-associated metabolite class expression profiles (Fig. S4, *A* and *B*) with BDQ eliciting maximum number of changes in metabolism to facilitate Mtb’s survival in the presence of sub-inhibitory doses of antibiotics.

### DPA identifies selective modulation of carbon and nitrogen metabolites by various drugs

**Figure 5:**
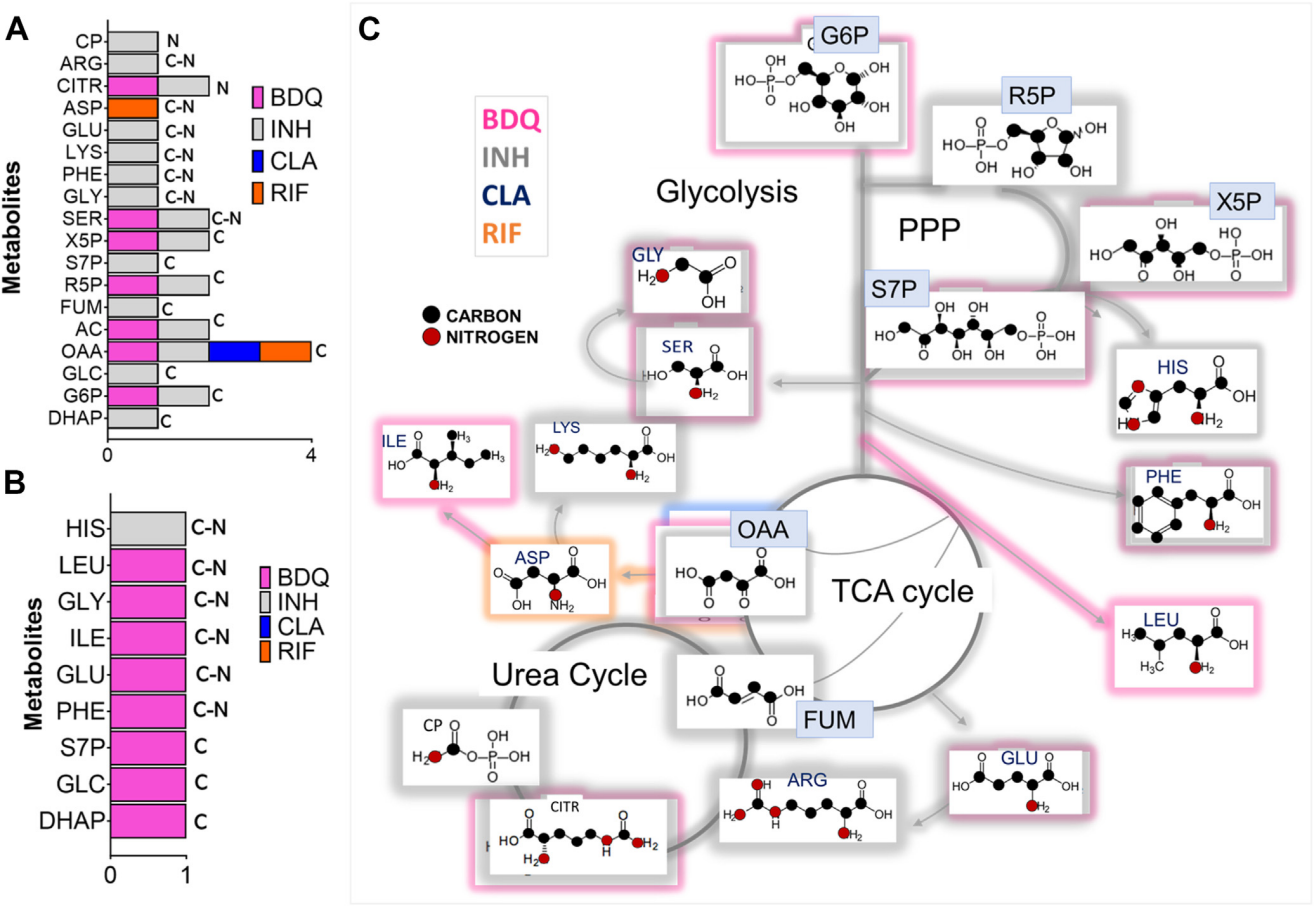
**Central carbon and nitrogen metabolites that are up and downregulated in Mtb treated with different drugs.***A*, upregulated metabolites. *B*, downregulated metabolites are color coded according to the drugs BDQ, INH, RIF and CLA. *C*, overlay of the C, N and C-N metabolites in the various metabolic pathways that they are biosynthesized. DPA datasets were generated using experimental RNA-seq data from three biological replicates of Mtb cultures grown in the presence of drugs for 24 h. The X-axis on (*A*) and (*B*) represents either the presence (assigned a value of 1) or absence (assigned a value of 0) of a particular metabolite that is up or downregulated in the presence of drugs.

In this section, we focus on the central carbon and nitrogen metabolites that are reprogrammed in Mtb in response to drugs. Carbon and nitrogen metabolic fluxes are core to sustaining cellular growth and function. In Figure 5, *A* and *B* we have shown drug-associated differentially expressed metabolites that are involved in the central carbon and nitrogen metabolism of Mtb. Figure 5*C* is an overlay of the up and downregulated metabolites and the metabolic pathways that are involved. The CCM metabolites upregulated by INH and BDQ are grouped into carbon (C), nitrogen (N) and C-N (carbon-nitrogen) metabolism (Fig. 5*A*). The C, N and C-N metabolite expressions during drug treatment was identified using DPA of the RNA-seq data, which was not straightforward from the direct analysis of RNA-seq data alone.

Metabolites involved in C metabolic pathways include glycolytic intermediates: glucose 6-phosphate (G6P), dihydroxyacetone phosphate (DHAP), glucose (GLC); TCA and glyoxylate cycle intermediates: OAA, fumarate (FUM), acetate (AC); non-oxidative PPP intermediates: xylulose 5-phosphate (X5P), sedoheptulose 7-phosphate (S7P), ribose 5-phosphate (R5P). Metabolites involved in N and C-N metabolic pathways including carbamoyl phosphate (CP), citrulline (CITR), arginine (ARG), glutamate (GLU), lysine (LYS), phenylalanine (PHE), glycine (GLY) and serine (SER) were upregulated in INH-treated cells. CITR, ARG, CP, GLU and FUM are intermediates of the urea cycle that was upregulated by INH (Fig. 5*C*). GLY and SER are derived from glycolytic intermediates; an upregulated series of metabolites in glycolysis and glycolysis-derived amino acids suggest that growth at sub-inhibitory concentrations of INH results in a higher C and C-N flux through this pathway. In comparison to INH, BDQ had a limited number of metabolites that were upregulated. This was because certain metabolites including GLY, S7P, GLU were identified as both up and downregulated in the presence of BDQ. However, the ranking of these metabolites in the up- and downregulated classes were significantly different. We used the ranking criteria to assign a metabolite as up or downregulated: for instance, the rank of AC1PIM2 in the upregulated class is 79, and the rank is 2 within the downregulated class. Hence AC1PIM2 was categorized as downregulated in BDQ-treated cells (Supplementary File 2).

OAA, the metabolic intermediate of the TCA cycle, anaplerosis, and gluconeogenesis was the only metabolite upregulated (targeted) by all four drugs at sub inhibitory concentrations. To our knowledge, this is the first report identifying OAA as one of the reprogrammed nodes during growth of Mtb in all four drugs. We checked the normalized counts for eight genes that are involved in OAA metabolism (Fig. S5). Rv1131 encoding for methylcitrate synthase (PrpC) that forms methycitrate from propionyl-CoA and OAA was upregulated in all four drugs. Malate dehydrogenase (Rv1240), probable malate:quinone oxidoreductase (Rv2852c) and pyruvate carboxylase (Rv2967c) had increased expression levels in CLA, BDQ and RIF-treated cells. These results highlight the power of DPA in providing new insights into the significance of a metabolic node or reaction which was not identified by analyzing the RNA-seq data alone. Aspartate (ASP) was upregulated only in RIF. Figure 5*B* identifies drug-associated downregulated C and N metabolites. BDQ downregulated C pathway intermediates DHAP, GLC and S7P and amino acids SER, isoleucine (ILE), GLY and leucine (LEU). INH downregulated histidine (HIS). It is evident that Mtb cells growing in the presence of BDQ selectively upregulated glycolytic, TCA cycle and PPP metabolites and downregulated several amino acids. Whilst exposure to INH upregulated central metabolic intermediates, accompanied by the downregulation of amino acid HIS.

In summary, we have demonstrated a powerful application of our DPA platform to interrogate the RNA-seq data of Mtb during survival on various drugs. Here we provide new information on expression profiles of metabolites involved in CCM, energy, cofactor, mycolic acid, lipid and amino acid pathways that were implicated in Mtb exposed to various drugs at subinhibitory concentrations. Our work identifies metabolic adaptations that are important for Mtb to persist and resist drugs.

## Discussion

Metabolic physiology is important for drug resistance, tolerance and persistence in Mtb. We and others have demonstrated that Mtb flexibly uses metabolic fluxes to co-catabolize various nutrients to fuel intracellular growth and to withstand environmental stresses such as host defenses and exposure to drug treatment (16, 21, 27). In this study, we investigated the changes in central carbon and nitrogen metabolism of Mtb exposed to the four anti-TB drugs including BDQ, INH, RIF and CLA. Although the main targets for these drugs are known, the underlying metabolic adaptations during drug treatment that contributes to Mtb’s survival, tolerance and resistance is not fully understood. This knowledge will help us to identify new targets that could provide opportunities for combinatorial and synergistic drug treatments for improving TB chemotherapy.

Genome-scale metabolic models allow systems wide evaluation of metabolic physiology, gene essentiality, and drug target discovery (21, 23, 28). Here we used our genome scale Mtb model GSMN-TB_aux for conducting DPA of RNA-seq data generated from Mtb exposed to various drugs. We present the first comprehensive report of central carbon and nitrogen metabolites that are implicated in Mtb growing in the presence of subinhibitory concentrations of the drugs. Genome scale models allow *in silico* assessments of growth, nutrient uptake and metabolism at large-scale which aids in designing optimal experiments to follow up on the predictions (23). GSMN-TB_aux was able to generate *in silico* biomass profiles which were similar to the experimentally derived CFU measurements providing evidence for biological relevance of genome scale models (Fig. 2). We conducted FBA and knockout analysis using GSMN-TB_aux to calculate metabolite abundances corresponding to the genes in the metabolic network. Metabolites are functional entities that are critical to sustaining biochemical reactions in a cell. RNA-seq data alone provided limited information about the metabolic changes (Supplementary File 3) and was not able to provide us with system-scale metabolite abundances associated with the gene expression. Therefore, we used DPA to conduct rank product analysis of metabolite abundances and mapped it to the RNA-seq profiles to derive the list of up and downregulated metabolites in Mtb exposed to various drugs (Fig. 3). To our knowledge, DPA is one of the very few platforms that facilitates direct mapping of RNA-seq (gene expression) data to metabolite signals.

Amongst the drugs tested, BDQ-treated Mtb cells had maximum number of downregulated metabolites involved in lipid and amino acid metabolism. This metabolite expression profile associated with drugs was significantly different to the previously reported metabolic response of Mtb in macrophages (21). Bonde *et al.*, 2011 used DPA to map up- and downregulated metabolites of Mtb in a macrophage environment and demonstrated downregulation of metabolites in central metabolism with upregulation of cell wall components. In contrast, our results show upregulation of central metabolism and mycolic acids in BDQ and INH exposed cells. Mtb was predicted to alter its biomass composition with an increase in the cell wall and triacylglycerol metabolites under hypoxic and anaerobic conditions (27). AA were shown to be upregulated in Mtb growing inside human macrophages (21). Here we found a different trend of AA metabolism on drugs; AA classes were significantly downregulated on exposure to BDQ. The differences observed between ours and Bonde *et al.*, 2011 (21) highlights that the metabolic response in Mtb to a macrophage environment is strikingly different to its response to drugs. As expected, there are differences in types of stress generated by macrophages and drugs on Mtb. The differences between our work and others can be explained by different research questions asked and different experimental setups where, in this study we used subinhibitory concentrations of drugs to allow Mtb’s growth as opposed to a macrophage environment that is growth restrictive, and nutrient limited for Mtb.

BDQ and INH upregulated common metabolites that were intermediates for glycolysis, TCA cycle and PPP (Fig. 4). Resveratrol, a polyphenol, that inhibited ATP synthase activity in *E. coli* and *Salmonella typhimurium* also affected central metabolic pathways such as glycolysis and the TCA cycle, leading to imbalances in bacterial energy metabolism (35, 36). Our findings agree with previous reports on bacterial metabolic remodeling that was required to survive drug-induced stress. Mtb treated with BDQ relied on glycolysis for ATP production; therefore, targeting both energy production by BDQ and inhibition of glycolytic pathway led to rapid of killing of Mtb (15, 18).

OAA was the only metabolite that was commonly upregulated across all four drugs (Figs. 4, 5). This was not surprising as a previous metabolomic study demonstrated that Mtb adopted a similar metabolic profile during exposure to different antibiotics (17). Although different drugs or antibiotics have distinct molecular and cellular targets, the underlying central metabolic responses or adaptations that are a consequence of the effects and are needed to survive are common cross distinct drugs. OAA is a TCA cycle intermediate that is also a critical node for gluconeogenesis and anaplerotic reactions. Anaplerotic metabolism of OAA involving enzyme phosphoenol pyruvate carboxykinase is important for Mtb’s survival in the host and is an attractive drug target in Mtb (34, 37). In addition to OAA, FUM and AC were upregulated in INH-treated cells, suggesting the likely use of a reductive TCA cycle by Mtb during exposure to INH. Using *in silico*-based modeling of gene expression data Fang *et al.*, 2012 identified a similar switch from oxidative to a reductive TCA cycle by Mtb during growth under hypoxia (27). RIF, streptomycin (STREP), and INH have been demonstrated to activate Mtb’s isocitrate lyase that replenishes TCA cycle intermediates with a significant increase in the pool sizes of pyruvate, succinate, malate, and FUM (17). Our work demonstrated a similar metabolic response of Mtb to INH with increases in FUM and pyruvate. Additionally, we show OAA as a common target across RIF, CLA, INH, and BDQ, which has not been reported in the previous work. The differences in our work vs. Nandkumar *et al.*, 2014 (17) could be attributed to the different omic techniques and experimental setups including the MICs used to measure metabolic responses. Nevertheless, the two studies are complementary and show upregulation of the TCA cycle intermediates in Mtb exposed to drugs. OAA and other carbon sources such as cholesterol, acetate, and pyruvate that allow flexible use of anaplerotic, gluconeogenesis, and TCA cycle pathways help Mtb to survive at an acidic pH of 5.7; this is a strategy adopted by Mtb to resist host hostile environment (33). *Entamoeba histolytica*, the causative agent of parasitic infections in humans, was found to resist oxidative stress using OAA which helped the parasite to survive in the large intestine (32). The same study also demonstrated the involvement of OAA in *Caenorhabditis elegans* against oxidative stress. Thus, OAA is an important metabolite for drug-induced stress responses in Mtb.

AA C-N metabolism is important in Mtb for intracellular growth and replication (Borah *et al.*, 2019); here we expand our understanding of which AAs are remodeled in Mtb on exposure to drugs. We observed a selective up and downregulation of AAs by different drugs (Fig. 5). While upregulation of central carbon metabolic intermediates was a common trend in various drugs tested, the changes in AA profiles was drug specific. GLU and the urea cycle intermediates ARG, CITR, CP were upregulated in INH-treated Mtb cells. GLU is the central N metabolic in Mtb and is one of the primary N sources acquired by Mtb during intracellular replication (38, 39). In addition to metabolism, GLU also plays important physiological roles in neutralizing cytoplasmic acidic pH generated through the consumption of propionate through the methyl citrate cycle in Mtb (40). Perturbation of ARG biosynthesis elicited kanamycin tolerance and persistence *in M. smegmatis* through upregulation of the transcriptional regulator WhiB7 (41). In our work, we similarly demonstrate modulation of ARG levels in response to INH treatment. SER biosynthesis is critical for Mtb’s N metabolism and survival in the host. Deletion of Rv0884c (phosphoserine transaminase, SerC) reduced intracellular survival of Mtb demonstrating this gene as a potential drug target (38). SER was upregulated in both BDQ and INH-treated cells suggesting the involvement of SER in drug tolerance and survival. Here we have shown key metabolic intermediates in C and N metabolism including glycolytic and TCA cycle-derived intermediates and amino acids GLU, SER and ARG that are implicated in Mtb exposed to subinhibitory concentration of the drugs. Our analysis highlights genes: PrpC, Mdh, Mqo and Pca involved in OAA metabolism and SerC involved in SER metabolism as targets for exploring new inhibitors and their synergistic effects with BDQ, RIF, CLA and INH. Our work provides directions for developing synergistic therapies that will help to fight drug-resistance in TB.

In summary, here we applied DPA to interrogate RNA-seq data sets and derived drug-associated metabolite expression profiles in Mtb. The DPA workflow can be extended to investigate RNA-seq datasets from any biological system that can be represented as a genome scale metabolic network. The drug-associated profiles provide us with an understanding of how Mtb adapts its metabolism to survive in the presence of drugs providing avenues for developing combinatorial therapeutics for TB.

## Experimental procedures

### Growth media and bacterial strains used

*M. tuberculosis* (Mtb) H37Rv mc^2^6260 (*ΔleuCD ΔpanCD*) kindly provided by Prof William R. Jacobs Jr was used as the model strain. Mtb mc^2^6260 were grown in Middlebrook 7H9 (Difco) supplement with 10% oleic acid-albumin-dextrose-catalase (OADC) (Thermo Fisher), 0.2% (v/v) glycerol (Fisher Scientific) and 0.05% (v/v) Tween 80 (Fisher Scientific) and extra supplements of 50 ug/ml leucine (Sigma Aldrich) and 24 μg/ml pantothenate (Sigma Aldrich). Solid media cultures for Mtb H37Rv mc^2^6260 were grown on Middlebrook 7H11 agar (Difco) supplemented with 10% OADC, 0.5% (v/v) glycerol, 50 μg/ml leucine, and 24 μg/ml pantothenate. The bacterial strain and the growth condition stated here was from previously published work (31).

### Minimum inhibitory concentration determination

The MIC assay and results were generated in previously published work (31). Rifampicin, isoniazid, clarithromycin, and bedaquiline were purchased from Sigma and were prepared as per the manufacturer’s notes. To mitigate the overwhelming stress responses, Mtb cells were exposed to sub-inhibitory concentration (one-fourth times MIC) of drugs. Minimal inhibitory concentration (MIC) was conducted using a microarray broth dilution assay (31). Briefly, log phase bacteria broth culture was diluted to 10^5^ colony forming units (CFU)/ml and incubated with drugs in 96-well plates for 7 days. The MIC was determined as the lowest concentration of drugs that shows no increase of OD_600_ (see Table S1).

### RNA-seq analysis

The RNA-seq data analyzed in this work, and the methods used to generate the data have been previously published (31). Transcriptome sequencing of Mtb H37Rv mc^2^6260 after drug treatments was performed after 24 h of incubation with the drugs. Briefly, the log phase of Mtb H37Rv mc^2^6260 cells was exposed to drugs for 24 h. Total RNA was extracted using Trizol/chloroform assay and then cleaned up with RNA clean and concentrator kit (Zymo Research) following the manufacturer’s instruction. DNAse treatment was performed to remove any contaminating DNA. Ribosomal RNA was removed from total RNA using NEBNext rRNA Depletion (bacteria) (NEB). NEBNext Ultra II RNA Library Prep Kit for Illumina (NEB) was used for sequencing library preparation as instruction, including RNA fragmentation, first and second-stand cDNA synthesis, adapter ligation, indexing, and library fragment PCR enrichment. RNA library was performed using 2 × 150 pair-end sequencing on the Illumina NovaSeq 6000 instrument. Sequencing raw data was generated by the NovaSeq Control Software on the instrument and converted to fastq files. Quality control of fastq files was performed using fastQC (Version 0.11.9). Adapters were removed from Cutadapt (Version 4.0) and checked with fastQC to confirm complete removal. Reads were aligned to Mtb H37Rv reference genome (NC_000962.3) using Bowtie2 (Version 2.5.0). Mapping was checked with QualiMap (Version 2.2.2 days) and reads were counted using featureCounts. Genes counts were normalized to sequencing depth using edgeR. Differentially expressed genes were calculated using Limma-Voom. *p*-values were calculated based on the mean-variance values of log2 fold change and adjusted using the Benjamini and Hochberg method. Genes with a log2 fold change >1 or < -1 and an adjusted *p*-value of <0.05 were considered significant. All bioinformatics analyses were performed using the Galaxy web platform (31).

### Genome-scale metabolic modeling and flux balance analysis (FBA)

GSMN-TB_2 was used as the reference genome-scale model for constructing GSMN-TB_aux, which is auxotrophic for leucine and pathothenate due to the deletions of Rv3602c (*panC*), Rv3601c (*panD*), Rv2988c (*leuC*) and Rv2987c (*leuD*) (42). This strain referred to as mc^2^6206 is a double auxotroph, fully antibiotic-sensitive biosafety level 2 Mtb strain (43). GSMN-TB_2 was modified: reactions catalyzed by PanCD and leuCD were inactivated to represent Mtb ΔleuCD ΔPanCD double auxotrophic strain that was used for RNA-seq analysis. Uptake reactions R942 and R943 were added to allow uptake of pantothenate and CBHCAP (3-carboxy-2-hydroxy-4-methylpentanoate) from the external media (See problem file in Supplementary File 1). Reactions R222, R223, and R224 use CBHCAP as a substrate for the production of leucine, so leucine gates were not opened in the media. FBA was used to interrogate GSMN-TB_aux and to obtain the lists of genes associated with the synthesis of metabolites in the network. The problem file for FBA was formulated to have glycerol and tween-80 as carbon sources, glutamine as nitrogen sources, and trace elements.

### Differential producibility analysis (DPA)

The goal of DPA is to identify metabolites that are significantly affected by the widespread changes in gene transcription. The pre-requisites for DPA are the fold changes in gene expression derived from transcriptomics, considering the metabolites produced as a consequence of gene up-regulation or down-regulation (21). DPA analyses metabolites within the context of the entire metabolic network. Differential transcriptomics data of multiple drug-regimens were analyzed to identify characteristic global metabolic changes using the GSMN-TB_aux. To perform DPA, we first generated an FBA-derived metabolite producibility plot to systematically identify the set of genes involved in the production of each metabolite. In this process, the production of each metabolite was maximized and set as the “objective function.” Next, for each metabolite, contribution of gene(s) affecting its production is calculated using “median metabolite signal.” This step provided insight into the specific gene(s) involved in the synthesis of individual metabolites and enabled us to perform rank analysis. Here, we have used FBA for biomass production as the starting step followed by DPA of individual metabolites where in objective function was ‘metabolite production’.

DPA was performed using GSMN-TB_aux model to convert transcriptomics data to metabolite abundance data *via* FBA, with an objective of identifying the metabolic reactions affected by the experimental conditions. DPA was performed using RNA-seq data obtained from known drug treatments, *viz.*, BDQ, RIF, INH, and CLA with respect to the control (untreated/no drug). Venn diagrams were generated using the tool available at: https://bioinformatics.psb.ugent.be/webtools/Venn/(last accessed: Aug 01, 2024). In-house scripts for performing iterative FBA and DPA analysis were written in Perl version v5.34.0. R version 4.1.2 and package RankProd were used to perform the Rank Product Analysis of the metabolites (44). All computations were performed on a 2 TB RAM Ubuntu 18.04.5 LTS server.

### Statistics

Statistics for the analysis of MIC data and survival ratio were performed using GraphPad Prism 10. RNA-seq analysis was performed as described in previous work (31). DPA statistics were performed using packages in Perl and R.

## Data availability

All relevant data are within the manuscript and its supporting information files.

## Supporting information

This article contains supporting Information (31).

## Conflict of interest

The authors declare that they have no conflicts of interest with the contents of this article.
