# Supplementary material for: Differential producibility analysis reveals drug-associated carbon and nitrogen metabolite expressions in Mycobacterium tuberculosis
Source: J Biol Chem. 2025 Feb 8;301(3):108288. doi: 10.1016/j.jbc.2025.108288 (PMC11986224; doi:10.1016/j.jbc.2025.108288)
Supplement: Table S1 [file mmc1.docx]

Table S1 **MICs determined in this study**

|  | **Minimum Inhibitory Concentration (MIC) (µg/ml)** | **1/4^th^ MIC: Concentration used in this study (µg/ml)** |
| --- | --- | --- |
| Rifampicin (RIF) | 0.060 | 0.013 |
| Isoniazid (INH) | 1 | 0.250 |
| Clarithromycin (CLA) | 64 | 16 |
| Bedaquiline (BDQ) | 0.500 | 0.125 |
