# Supplementary material for: Differential producibility analysis reveals drug-associated carbon and nitrogen metabolite expressions in Mycobacterium tuberculosis
Source: J Biol Chem. 2025 Feb 8;301(3):108288. doi: 10.1016/j.jbc.2025.108288 (PMC11986224; doi:10.1016/j.jbc.2025.108288)
Supplement: Supplementary file information [file mmc2.docx]

**Supplementary file legends**

Supplementary File 1. Genome scale model network (GSMN-TB_aux) of Mtb showing all reactions, metabolite names and the problem file used for FBA.

Supplementary File 2. List of up and downregulated metabolites as identified by DPA.

Supplementary File 3. RNA seq datasets used for DPA analysis.

Supplementary File 4: Raw data sets used to generate survival plots (figure S1) and time-kill curves (figure S2).
