# Supplementary material for: Differential producibility analysis reveals drug-associated carbon and nitrogen metabolite expressions in Mycobacterium tuberculosis
Source: J Biol Chem. 2025 Feb 8;301(3):108288. doi: 10.1016/j.jbc.2025.108288 (PMC11986224; doi:10.1016/j.jbc.2025.108288)
Supplement: Supplementary figure and legend [file mmc3.docx]

Supplementary Figure S1

Supplementary Figure S1 **Survival ratio of Mtb after 48 hours’ treatment with RIF, BDQ, INH and clarithromycin (CLA) at the concentration of ¼, ½ , 1, 2 and 4 times of MIC**. MIC for RIF, BDQ, INH and CLAR is 0.06 μg/ml, 0,5 μg/ml, 1 μg/ml and 64 μg/ml. Survival ratio was determined by comparing culture’s CFUs at indicated time point to the CFUs of starter inoculum (10^7^ CFU/mL). Data sets used to generate this graph are provided in supplementary file 4. Each point represents mean ± SEM for at least three biological replicates.

Supplementary Figure S2


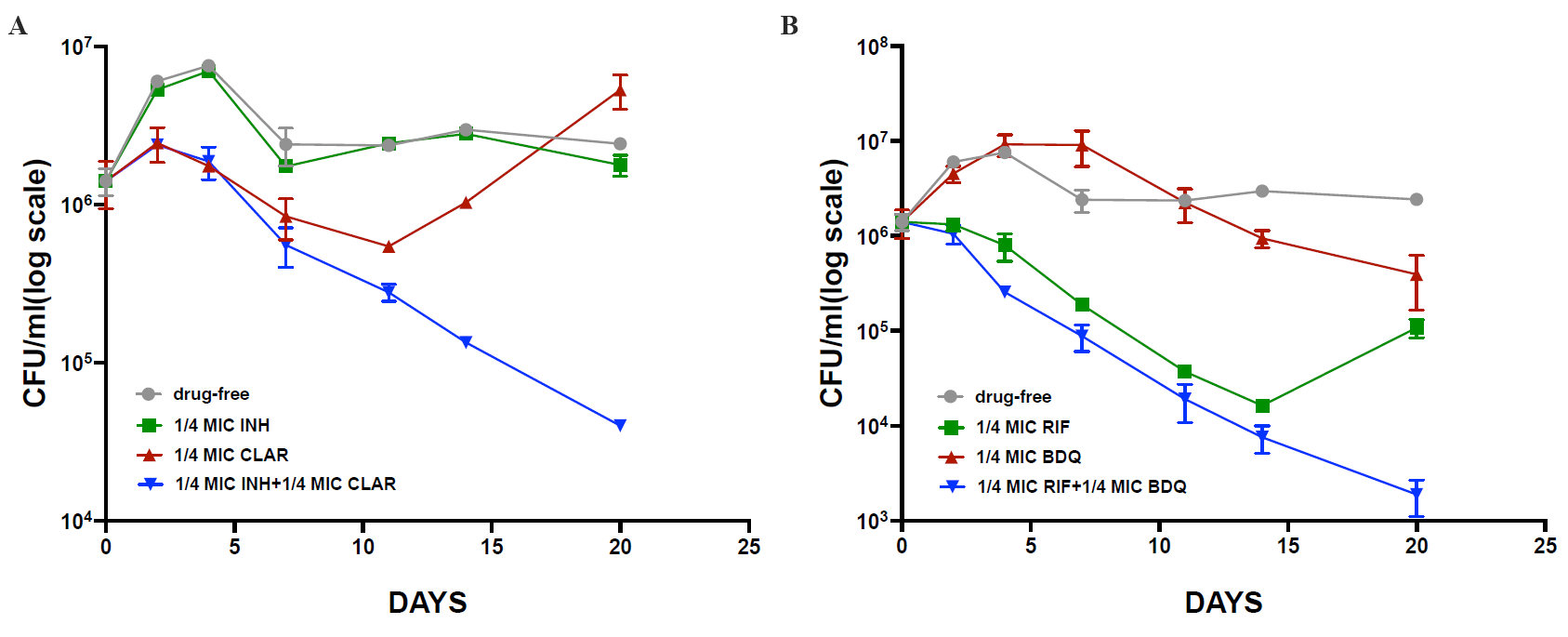


Supplementary Figure S2 **Time-kill curves of Mtb exposed to various drugs**. The CFU numbers are calculated for Mtb exposed to 1/4^th^ MICs of drugs or drug combinations over a period of time of up to 20 days. The time-kill curves show that 1/4^th^ MIC of individual drug combinations exert bacterial growth inhibitions but limited cellular stress that allowed RNA-seq and metabolic analyses of drug-treated Mtb. Each point represents mean ± SEM for at least three biological replicates.

Supplementary Figure S3


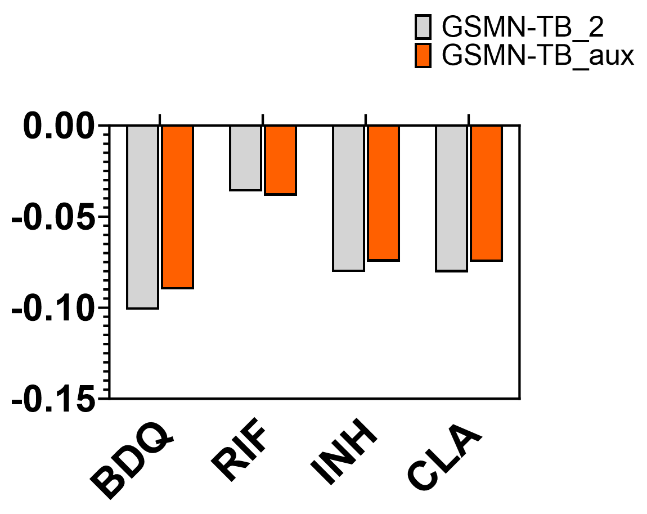


Supplementary Figure S3. **Comparison of biomass predictions by GSMN-TB_aux and GSMN-TB_2**. Data shows log 2-fold change of biomass production calculated using FBA of GSMN-TB_aux and GSMN-TB_2 in growth media with various drugs. There were no significant differences between the two model predictions and the fold change compared to control followed similar distribution pattern for the two models with BDQ exerting the maximum inhibition. Data are shown for the four drugs compared to the control (untreated/no drug) condition. Biomass was predicted for Mtb exposed to ¼^th^ MICs of BDQ, RIF, INH and CLA using 7H9 as the base media (see methods for details for *in silico* media formulation).

Supplementary Figure S4


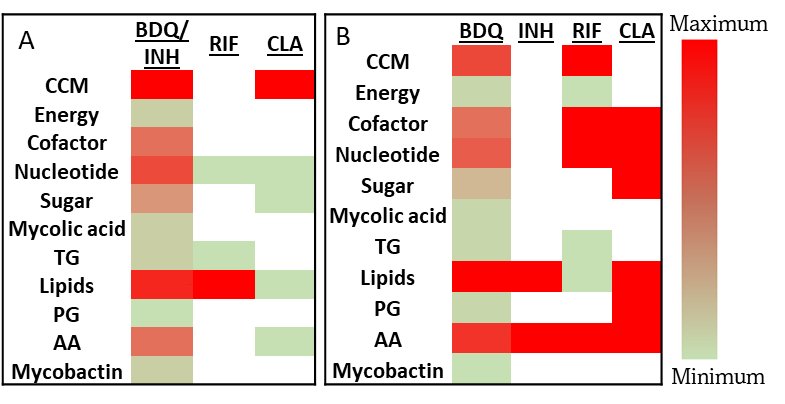


Supplementary Figure S4. **DPA generated metabolite expression profiles associated with BDQ, INH, RIF and CLA**. A) Upregulated metabolite expression patterns. B) Downregulated metabolite expression profiles. DPA datasets were generated using experimental RNA-seq data from three biological replicates of Mtb cultures grown in the presence of drugs for 24 hours. The blank cells in A and B indicate no changes in metabolite expressions under those conditions.

Supplementary Figure S5


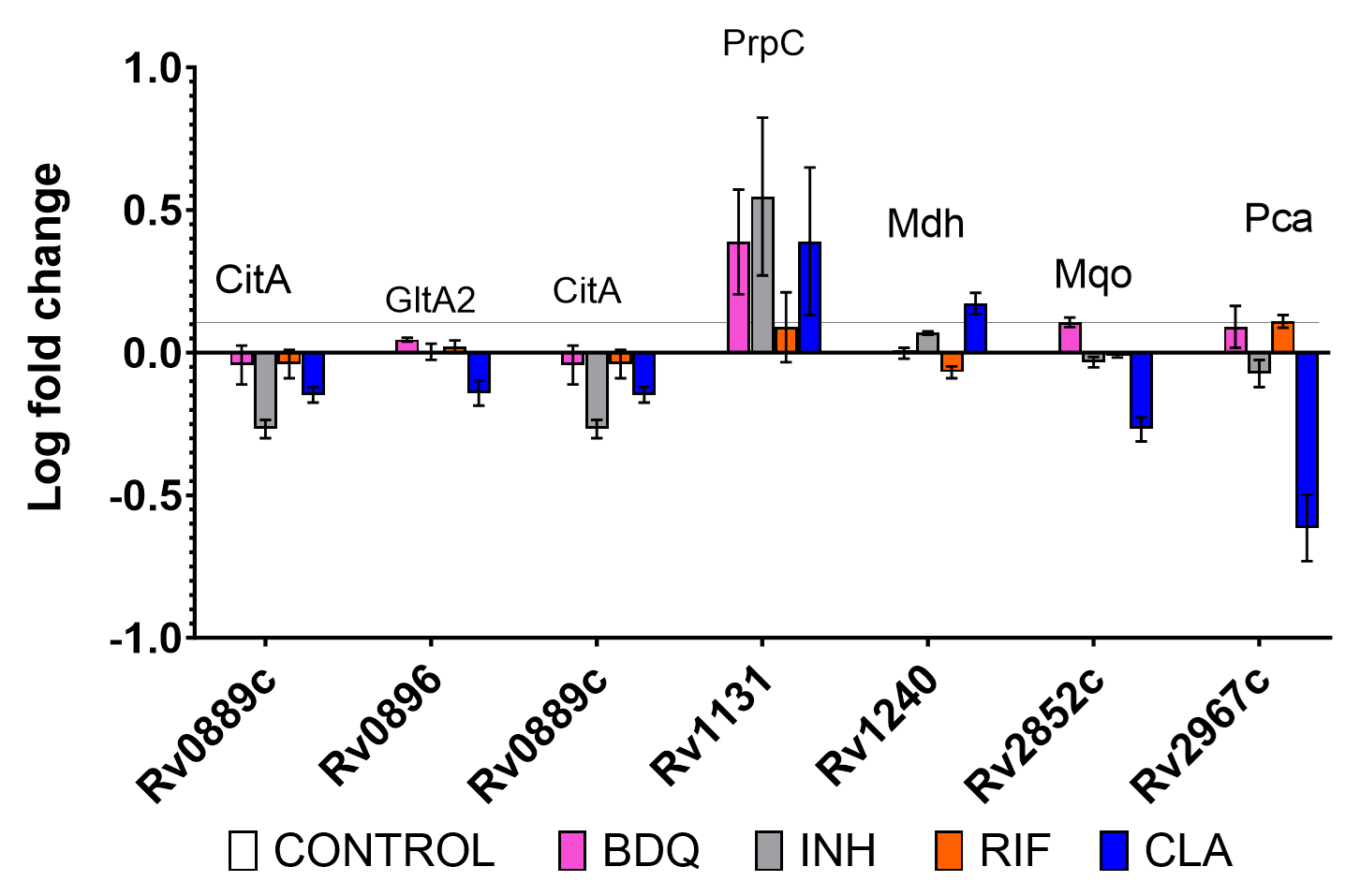


Supplementary Figure S5: **Gene counts of drug treated Mtb vs the control**. Data shown are normalized gene counts derived from RNA-seq experiments of control (untreated) and BDQ, RIF, CLA and INH drug-treated Mtb. A threshold of 0.1 was set to identify changes in expression of genes. The genes analysed in this graph participate in the biochemical reactions involving oxaloacetate as an intermediate. Rv0889c: citrate + CoA = acetyl-CoA + H2O + oxaloacetate; Rv0896: citrate + CoA = acetyl-CoA + H2O + oxaloacetate; Rv0889c: citrate + CoA = acetyl-CoA + H2O + oxaloacetate; Rv1131: propionyl-CoA + oxaloacetate + H2O = 2-methylcitrate + CoA; Rv1240: catalytic activity: (S)-malate + NAD+ = oxaloacetate + NADH; Rv2852: (S)-malate + acceptor = oxaloacetate + reduced acceptor; Rv2967: ATP + pyruvate + HCO(3)(-) = ADP + phosphate + oxaloacetate. Values were calculated using three biological replicates ± standard error of the mean.
